# Antepartum SARS-CoV-2 infection and adverse birth outcomes in South African women

**DOI:** 10.7189/jogh.12.05050

**Published:** 2022-12-03

**Authors:** Marta C Nunes, Stephanie Jones, Renate Strehlau, Vuyelwa Baba, Zanele Ditse, Kelly da Silva, Lané Bothma, Natali Serafin, Vicky L Baillie, Gaurav Kwatra, Megan Burke, Amy Wise, Mary Adam, Philiswa Mlandu, Mpolokeng Melamu, Juliette Phelp, Wendy Fraser, Colleen Wright, Elizabeth Zell, Yasmin Adam, Shabir A Madhi

**Affiliations:** 1 South African Medical Research Council, Vaccines & Infectious Diseases Analytics (VIDA) Research Unit, Faculty of Health Sciences, University of the Witwatersrand, Johannesburg, South Africa; 2 Department of Science and Technology/National Research Foundation, South African Research Chair Initiative in Vaccine Preventable Diseases, Faculty of Health Sciences, University of the Witwatersrand, Johannesburg, South Africa; 3 Nkanyezi Research Unit sub-division of VIDA, Department of Paediatrics and Child Health, School of Clinical Medicine, Faculty of Health Sciences, University of the Witwatersrand, Johannesburg, South Africa; 4 Department of Obstetrics and Gynecology, Chris Hani Baragwanath Academic Hospital, Faculty of Health Sciences, University of the Witwatersrand, Johannesburg, South Africa; 5 Department of Obstetrics and Gynecology, Rahima Moosa Mother and Child Hospital, Faculty of Health Sciences, University of the Witwatersrand, Johannesburg, South Africa; 6 Lancet Laboratories, Johannesburg, South Africa; 7 Division of Anatomical Pathology, School of Pathology, Faculty of Health Sciences, University of the Witwatersrand, Johannesburg, South Africa; 8 Stat-Epi Associates, Inc., Ponte Vedra Beach, Florida, USA; 9 African Leadership in Vaccinology Expertise, Faculty of Health Sciences, University of the Witwatersrand, Johannesburg, South Africa

## Abstract

**Background:**

SARS-CoV-2 infection in pregnant women has been associated with severe illness in the women and higher rates of premature delivery. There is, however, paucity of data on the impact of the timing of SARS-CoV-2 infection and on symptomatic or asymptomatic infections on birth outcomes. Data from low-middle income settings is also lacking.

**Methods:**

We conducted a longitudinal study from April 2020 to March 2021, in South Africa, where symptomatic or asymptomatic pregnant women were investigated for SARS-CoV-2 infection during the antepartum period. We aimed to evaluate if there was an association between antepartum SARS-CoV-2 infection on birth outcomes. SARS-CoV-2 infection was investigated by nucleic acid amplification test (NAAT), histological examination was performed in a sub-set of placentas.

**Results:**

Overall, 793 women were tested for SARS-CoV-2 antenatally, including 275 (35%) who were symptomatic. SARS-CoV-2 infection was identified in 138 (17%) women, of whom 119 had symptoms (COVID-19 group) and 19 were asymptomatic. The 493 women who were asymptomatic and had a negative SARS-CoV-2 NAAT were used as the referent comparator group for outcomes evaluation. Women with COVID-19 compared with the referent group were 1.66-times (95% confidence interval (CI) = 1.02-2.71) more likely to have a low-birthweight newborn (30% vs 21%) and 3.25-times more likely to deliver a very low-birthweight newborn (5% vs 2%). Similar results for low-birthweight were obtained comparing women with SARS-CoV-2 confirmed infection (30%) with those who had a negative NAAT result (22%) independent of symptoms presentation. The placentas from women with antenatal SARS-CoV-2 infection had higher percentage of chorangiosis (odds ratio (OR) = 3.40, 95% CI = 1.18-.84), while maternal vascular malperfusion was more frequently identified in women who tested negative for SARS-CoV-2 (aOR = 0.28, 95% CI = 0.09-0.89).

**Conclusions:**

Our study demonstrates that in a setting with high HIV infection prevalence and other comorbidities antenatal SARS-CoV-2 infection was associated with low-birthweight delivery.

The current coronavirus disease 2019 (COVID-19) pandemic, caused by the severe acute respiratory syndrome coronavirus-2 (SARS-CoV-2), was declared a global health emergency in March 2020. By June 30, 2022, 12.1 million SARS-CoV-2 infections have been reported in Africa, of which 4.0 million cases are from South Africa [1]. Pregnant women are at similar risk as the general population for being infected by SARS-CoV-2 [2,3], albeit at higher risk of progressing to severe disease. In pregnant women COVID-19 may lead to adverse pregnancy outcomes, and the virus could transmit to the foetus or newborn [4,5]. Several clinical studies, analyses from national surveillance systems and systematic reviews have been published on COVID-19 in pregnant women. Nevertheless, studies evaluating the impact of SARS-CoV-2 infection on birth outcomes in African women are sparse, and mainly describe retrospective analyses or without contemporaneous control group of SARS-CoV-2 uninfected pregnant women [6-11].

SARS-CoV-2 infection during pregnancy has been associated with approximately a 2-fold increased risk of hospital admission, maternal admission to an intensive care unit (ICU) and invasive ventilation, compared with non-pregnant women [2,11-13]. Pregnant women with COVID-19 have also been reported to have a higher case fatality risk compared with non-infected pregnant women [11,14,15]. Risk-factors associated with severe COVID-19 in pregnant women are similar to those in non-pregnant adults [2,3,12,16-18].

SARS-CoV-2 infection during pregnancy has also been associated with a higher risk of preterm delivery, stillbirth, low-birthweight and lower Apgar scores at birth [2,8,15,17,19]. Nevertheless, since SARS-CoV-2 infection varies phenotypically from asymptomatic infection or mild respiratory illness to hospitalization with multiorgan failure and death [20], the severity of disease and the timing of infection during pregnancy may affect foetal outcome.

## METHODS

### Study design

We conducted a longitudinal cohort study of pregnant women investigated for SARS-CoV-2 infection during the antepartum period to assess any association of SARS-CoV-2 infection and adverse birth outcomes.

Pregnant women were screened for participation and enrolled from April 17, 2020 to September 18, 2020 which coincided with the first wave of the COVID-19 pandemic in South Africa. Follow-up for pregnancy outcomes continued until March 17, 2021. Women were enrolled at two public hospitals in the Johannesburg region where health care is provided at no cost to pregnant women and children: Chris Hani Baragwanath Academic Hospital (CHBAH), a tertiary care hospital, located in Soweto, and Rahima Moosa Mother and Child Hospital (RMMCH), a secondary district hospital located in Coronationville. The prevalence of HIV infection in antenatal attendees at the two facilities is approximately 28% at CHBAH and 19% at RMMCH. During the first wave of COVID-19 in South Africa, hospital protocols were established to triage pregnant women presenting with respiratory symptoms, where women managed as outpatients were seen in designated areas and those requiring hospitalization were admitted to isolation wards. Study-staff undertook active surveillance in the antenatal and hospital wards to identify pregnant women who presented with symptoms suggestive of COVID-19 (cough, sore throat, fever/feeling feverish, chest pain/shortness of breath, rhinitis, ageusia, anosmia, myalgia, arthralgia, headache or diarrhoea) for possible study enrolment and SARS-CoV-2 testing. In addition, asymptomatic women attending routine antenatal care were approached, enrolled, and tested for SARS-CoV-2 with maximum of 10 per day during weekdays. Women who consented to study participation were followed-up until delivery by regular telephonic contact and requested to contact the site when in labour. Study-staff also screened all women presenting in labour at the hospitals to identify those who had been enrolled antenatally. Women who delivered outside of the study-hospitals, or when no study-staff were on duty, had information on outcomes abstracted following review of their medical records or by interview.

The main outcome was to evaluate the association of SARS-CoV-2 infection diagnosed by nucleic acid amplification test (NAAT) in pregnant women with symptomatic illness (ie, COVID-19 group) and adverse birth outcomes of spontaneous preterm delivery (<37 weeks gestational age), low-birthweight (<2500 g), very low-birthweight (<1500 g) or intrauterine foetal death. The referent group comprised of asymptomatic women at the time of enrolment with a negative NAAT result.

At enrolment, mid-turbinate nasal swabs were collected for investigation of SARS-CoV-2 infection by NAAT, as described [21]. NAAT was performed using the USA Centers for Diseases Control and Prevention Emergency use authorization assays to detect SARS-CoV-2 [22]. Samples were classified as positive for SARS-CoV-2 when both nucleocapsid targets (N1 and N2) were positive with a cycle threshold <40. Samples were classified as inconclusive if only a single target was detected.

Detailed information on demographics, health, pregnancy history and pregnancy complications were collected from all participants. At the time of delivery placentas were collected when possible. Placentas were placed in 10% buffered formalin and referred to the designated laboratory. Placental maternal and foetal surfaces were photographed before sampling according to standardised protocols and the macroscopic description recorded on a standardised template. Sections of the umbilical cord, membranes, and parenchyma were taken as recommended by the Amsterdam Placental Workshop Group Consensus protocols [23], and processed into paraffin embedded wax blocks. Subsequently, sections were cut, haematoxylin-eosin stained and produced for histological examination. Two dedicated pathologists (CW, WF), blinded to the SARS-CoV-2 infection status, examined the slides and reported on a standard template. The reported features were a summation of the macroscopic and microscopic features recorded. Additional details provided in the **Online Supplementary Document**.

SARS-CoV-2 NAAT was also undertaken on placental tissue from women identified with antenatal SARS-CoV-2 infection and a random selection of women who had a negative NAAT result. Prior to nucleic acid extraction, paraffin-embedded placental tissues were de-paraffinized using 0.5% Tween-20 followed by heating in a 650W microwave for 45 seconds. The samples were subsequently centrifuged, and the solid wax disc was removed [24]. The placenta tissue was digested using 10mg/mL proteinase K (Sigma, Poole, UK) and mechanical disruption methods [25]. Total nucleic acids were extracted from lysed placental samples and nasal swabs using the Bioer automated extraction system together with MagaBio plus Virus DNA/RNA purification kit II as per manufactures’ instructions (Hangzhou Bioer Technology Co. Ltd, China).

### Statistical considerations

Participants’ categorical characteristics were described as percentages and were compared between women with COVID-19 and the control group or women with positive and negative SARS-CoV-2 NAAT by χ^2^ or Fisher exact-test. Continuous variables were represented as mean or median and compared by Student’s *t* test or Mann-Whitney test, respectively. *P* values <0.05 were considered significant.

Study outcomes were described per newborn, with multiple birth pregnancies evaluated independently. A variable combining newborns with either low-birthweight or born preterm was generated (preterm/low-birthweight). Placental histological characteristics were compared between women with positive and negative NAAT results on nasal swab and restricted to women who had a singleton birth.

Two sub-analyses were performed, i) comparison of outcomes between overall women with positive and negative NAAT results during pregnancy; ii) comparison of outcomes between symptomatic and asymptomatic women at enrolment stratified by NAAT positivity.

Odds ratios (OR), both crude and adjusted (aOR), and 95% confidence intervals (CI) were estimated by logistic regression. Covariates were selected a priori based on the literature, and these included: maternal HIV infection status, comorbid conditions, pregnancy-related complications, previous premature births, and time between diagnosis and delivery (7 categories overall, from <7 days to >90 days). Associations between a positive NAAT or COVID-19 and adverse outcomes were considered statistically significant if the 95% CI did not cross 1. Study data were collected and managed through Research Electronic Data Capture [24]. Analyses were performed using STATA 13.1 (StataCorp, College Station, Texas 77845 USA) and SAS/STAT 9.4 (SAS Institute Inc., Cary, North Carolina, USA).

## RESULTS

**Figure 1:**
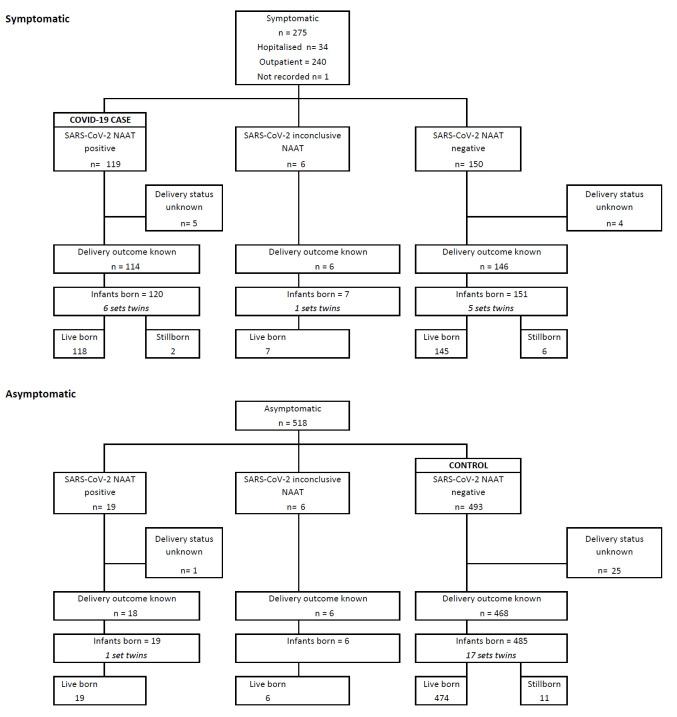
Participants’ flow diagram accordingly to symptoms presentation antenatally at the time of study enrolment.

Overall, 793 women were tested for SARS-CoV-2 infection antenatally, including 275 (35%) who presented with symptoms suggestive of COVID-19. Twelve women in whom the NAAT result was inconclusive were excluded from the outcome analyses (**Figure 1**). Overall, SARS-CoV-2 infection was confirmed in 138 (17%) women, including 119 of 275 (43%) presenting with symptomatic illness (COVID-19 group) and 19 of 518 (4%) who were asymptomatic. Among the 643 women in whom SARS-CoV-2 was not identified, 493 (77%) were asymptomatic (ie, referent group).

At enrolment, the mean age was 31 years, the mean gestational age was 31.5 weeks, with no difference based on NAAT positivity on nasal swab or between women in the COVID-19 group or control group. Women with a positive NAAT result compared with those with a negative test were more likely to have comorbidities (25% vs 16%, *P* = 0.015) and pregnancy-related complications (55% vs 43%, *P* = 0.023); and similarly, so for women in the COVID-19 and the referent groups (**Table 1**).

**Table 1:** Characteristics of women enrolled and tested for SARS-CoV-2 infection antenatally

	Overall n = 793	SARS-CoV-2 positive n = 138	SARS-CoV-2 negative n = 643	SARS-CoV-2 inconclusive n = 12	*P*-value*	Symptomatic and SARS-CoV-2 positive (COVID-19) n = 119	Asymptomatic and SARS-CoV-2 negative (control) n = 493	*P*-value†
Symptomatic at the time of enrolment					<0.001	-	-	
Yes	275 (35)	119 (86)	150 (23)	6 (50)				
No	518 (65)	19 (14)	493 (77)	6 (50)				
Admitted for respiratory condition at the time of enrolment					<0.001			<0.001
Yes	34 (4)	21 (15)	13 (2)	0 (0)		21 (18)	0 (0)	
No	758 (96)	116 (85)	630 (98)	12 (100)		97 (82)	493 (100)	
Missing	1	1	0	0		1	0	
Mean age in year at enrolment, SD	30.96.8	31.8, 6.6	30.7, 6.7	33.8, 11.5	0.08	31.8, 6.5	30.8, 6.6	0.16
Race					0.09			0.16
Black	758 (96)	129 (93)	620 (97)	9 (75)		112 (94)	479 (97)	
Asian	2 (0.3)	1 (1)	1 (0.2)	0 (0)		1 (1)	1 (0.2)	
Coloured	26 (3)	6 (4)	17 (3)	3 (25)		4 (3)	10 (2)	
Caucasian	4 (1)	1 (1)	3 (0.5)	0 (0)		1 (1)	2 (0.4)	
Other	1 (0.1)	1 (1)	0 (0)	0 (0)		1 (1)	0 (0)	
Missing	2	0	2	0		0	1	
Mean gestational age in weeks at enrolment, SD	31.5, 6.9	31.0, 7.6	31.6, 6.8	33.4, 6.4	0.39	30.9, 7.6	31.9, 6.6	0.14
Gestational age in weeks at enrolment					0.74			0.43
<34	405 (51)	73 (54)	327 (51)	5 (42)		64 (55)	238 (48)	
34-<37	168 (21)	26 (19)	141 (22)	1 (8)		22 (19)	111 (23)	
≥37	214 (27)	37 (27)	171 (27)	6 (50)		31 (26)	142 (29)	
<37	573 (73)	99 (73)	468 (73)	6 (50)		86 (74)	349 (71)	
Missing	6	2	4	0		2	2	
Median days between enrolment and delivery	30, 10-69	24, 6-72	31, 12-69	15, 4-57	0.11	25, 6-71	30, 12-66	0.38
IQR‡	(759)	(132)	(615)			(114)	(469)	
Multiple gestation pregnancy					0.35			0.42
Yes	30 (4)	7 (5)	22 (4)	1 (8)		6 (5)	17 (4)	
No	729 (96)	125 (95)	593 (96)	11 (92)		108 (95)	452 (96)	
Missing	34	6	28	0		5	24	
Previous premature births (<37 weeks)					0.022			0.09
Yes	141 (20)	29 (22)	109 (19)	0 (0)		11 (10)	24 (5)	
No	570 (80)	100 (78)	463 (81)	10 (100)		100 (90)	415 (95)	
Missing	82	9	71	2		8	54	
Previous miscarriages					0.35			0.42
Yes	30 (4)	7 (5)	22 (4)	3 (30)		6 (5)	17 (4)	
No	729 (96)	125 (95)	593 (96)	7 (70)		108 (95)	452 (96)	
Missing	34	6	28	2		5	24	
Previous stillbirths					0.82			0.79
Yes	52 (7)	9 (7)	43 (8)	0 (0)		8 (7)	35 (8)	
No	656 (93)	120 (93)	526 (92)	10 (100)		103 (93)	404 (92)	
Missing	85	9	74	2		8	54	
Comorbid condition§					0.015			0.005
Yes	136 (18)	34 (25)	102 (16)	0 (0)		29 (25)	68 (14)	
No	640 (82)	102 (75)	527 (84)	11 (100)		89 (75)	416 (86)	
Missing	17	2	14	1		1	9	
Living with HIV					0.26			0.19
Yes	208 (27)	31 (23)	176 (28)	1 (8)		25 (22)	134 (28)	
No	569 (73)	103 (77)	455 (72)	11 (92)		90 (78)	349 (72)	
Missing	16	4	12	0		4	10	
Syphilis serology					0.70			0.37
Reactive	11 (2)	1 (1)	10 (2)	0 (0)		0 (0)	8 (2)	
Non-reactive	689 (98)	121 (99)	556 (98)	12 (100)		105 (100)	422 (98)	
Missing	93	16	77	0		14	63	
Influenza vaccinated					0.13			0.07
Yes	30 (4)	8 (6)	20 (3)	2 (17)		7 (6)	13 (3)	
No	743 (96)	126 (94)	607 (97)	10 (83)		109 (94)	468 (97)	
Missing	20	4	16	0		3	12	
Tetanus toxoid vaccinated					0.61			0.12
Yes	447 (58)	75 (56)	365 (58)	7 (58)		61 (53)	290 (61)	
No	324 (42)	59 (44)	260 (42)	5 (42)		55 (47)	189 (39)	
Missing	22	4	18	0		3	14	
Pregnancy related complications‖					0.023			0.040
Yes	343 (45)	74 (55)	264 (43)	5 (42)		64 (55)	191 (41)	
No	422 (55)	60 (45)	355 (57)	7 (58)		52 (45)	280 (59)	
Missing	28	4	24	0		3	22	

SD – standard deviation, IQR – interquartile range

*P-values comparing women with a SARS-CoV-2 positive vs negative nucleic acid amplification test antenatally.

†*P*-values comparing symptomatic women with a SARS-CoV-2 positive nucleic acid amplification test vs asymptomatic women with a SARS-CoV-2 negative nucleic acid amplification test.

‡Numbers in parenthesis denote participants with available information.

§Comorbid conditions include: asthma, tuberculosis, chronic obstructive pulmonary disease/emphysema, other chronic lung disease, hypertension, cardiovascular disease, stroke, diabetes, anaemia, epilepsy, thyroid disease, cancer, malnutrition, obesity, renal failure, other organ failure.

‖Pregnancy related complication include: pregnancy induced hypertension, pre-eclampsia, eclampsia, HELLP (haemolysis, elevated liver enzymes, low platelet count), gestational diabetes, hyperemesis, trauma, pregnancy related infection, vaginal discharge, chorioamnionitis, maternal sepsis, maternal tachycardia, embolic disease, antepartum haemorrhage, postpartum haemorrhage, placenta previa/accrete/increta, placental abruption, premature contractions, premature rupture of the membranes, oligohydramnios, polyhydramnios.

Results are n (%) unless stated otherwise. Percentages calculated excluding missing values.

Pregnancy outcomes were available for 132 women identified with SARS-CoV-2 infection and 614 who had a negative NAAT result, who gave birth to 139 and 636 newborns’, respectively (**Figure 1**). Women with antepartum COVID-19 compared with the referent group were 1.66-times (95% CI = 1.02-2.71) more likely to deliver babies who were of low-birthweight (30% vs 21%) and 3.25-times (95% CI = 1.08-9.80) more likely of very low-birthweight (5% vs 2%). Similar observations were evident when restricting the analysis to women enrolled before 37 weeks gestational age (**Table 2**).

**Table 2:** Pregnancy outcomes in the control group and the COVID-19 group

	All study participants			
	**Symptomatic and SARS-CoV-2 positive (COVID-19)**	**Asymptomatic and SARS-CoV-2 negative (control)**		
	**n (%)**	**n (%)**	**OR (95% CI)**	aOR* (95% CI)
	**N = 120**	**N = 485**		
Live birth	118 (98)	474 (98)	Reference, 0.73 (0.16-3.34)	Reference, 0.86 (0.17-4.36)
Foetal death	2 (2)	11 (2)		
**Live births overall**				
	**N = 118**	**N = 474**		
Mode of delivery				
Vaginal	48 (41)	206 (44)	Reference	Reference
Caesarean-elective	25 (21)	101 (21)	1.06 (0.62-1.82)	1.27 (0.70-2.32)
Caesarean-emergency	45 (38)	167 (35)	1.16 (0.73-1.82)	0.98 (0.61-1.59)
Apgar score – 5 min				
0-3	1 (1)	2 (0.5)	2.00 (0.18-22.32)	2.39 (0.20-28.35)
4-6	2 (2)	9 (2)	0.89 (0.19-4.18)	0.87 (0.17-4.31)
7-10	107 (97)	429 (97)	Reference	Reference
Respiratory distress				
No	105 (91)	412 (89)	Reference	Reference
Yes	11 (9)	450 (11)	0.86 (0.43-1.72)	0.70 (0.34-1.44)
Gestational age in weeks at delivery				
≥37	87 (74)	390 (82)	Reference	Reference
<37	30 (26)	84 (18)	1.60 (0.99-2.58)	1.48 (0.89-2.46)
Birthweight in grams				
≥2500	81 (70)	365 (79)	Reference	Reference
<2500	34 (30)	95 (21)	**1.61 (1.02-2.55)**	**1.66 (1.02-2.71)**
<1500	6 (5)	9 (2)	**3.00 (1.04-8.68)**	**3.25 (1.08-9.80)**
Preterm/Low-birthweight				
No	74 (63)	351 (74)	Reference	Reference
Yes	43 (37)	123 (26)	**1.66 (1.08-2.54)**	**1.72 (1.08-2.68)**
**Live births, mothers enrolled at <37 weeks gestation**				
	**N = 85**	**N = 335**		
Gestational age in weeks at delivery				
≥37	55 (65)	251 (75)	Reference	Reference
<37	30 (35)	84 (25)	1.63 (0.98-2.71)	1.48 (0.80-2.74)
Birthweight in grams				
≥2500	54 (64)	244 (75)	Reference	Reference
<2500	30 (36)	81 (25)	**1.67 (1.00-2.79)**	1.52 (0.86-2,71)
<1500	6 (7)	9 (3)	**3.01 (1.03-8.82)**	3.15 (0.99-10.00)
Preterm/Low-birthweight				
No	46 (54)	226 (67)	Reference	Reference
Yes	39 (46)	109 (33)	**1.76 (1.08-2.85)**	1.69 (0.96-2.97)
**Live births, mothers enrolled at <34 weeks gestation**				
	**N = 63**	**N = 229**		
Gestational age in weeks at delivery				
≥37	39 (62)	162 (71)	Reference	Reference
<37	24 (38)	67 (29)	1.49 (0.83-2.66)	1.31 (0.57-2.99)
<34	13 (21)	32 (14)	1.69 (0.81-3.51)	1.05 (0.33-3.32)
Birthweight in grams				
≥2500	38 (60)	162 (73)	Reference	Reference
<2500	25 (40)	60 (27)	1.78 (0.99-3.19)	1.53 (0.75-3.11)
<1500	6 (10)	9 (4)	2.84 (0.95-8.47)	2.69 (0.74-9.82)
Preterm/Low-birthweight				
No	32 (51)	146 (64)	Reference	Reference
Yes	31 (49)	83 (36)	1.70 (0.97-2.99)	1.61 (0.77-3.37)

OR – odds ratio, aOR – adjusted odds ratio

*Odds ratio adjusted for HIV status (infected, uninfected), comorbid conditions (yes, no), pregnancy related complications (yes, no), previous premature births (yes, no), and time between diagnosis and delivery (categorical variable); for aOR, records numbers are less due to missing data in variables used for adjustment. Odds ratio in bold are statistically significant.

Comparing women with SARS-CoV-2 infection (ie, COVID-19 and asymptomatic infections) to those in whom NAAT was negative at enrolment, the rate of foetal death (1% vs 3%) was similar. Women with SARS-CoV-2 infection, however, had higher odds (aOR = 1.63, 95% CI = 1.04-2.55) of delivering a low-birthweight baby (30% vs 22%) (**Table 3**).

**Table 3:** Pregnancy outcomes by maternal SARS-CoV-2 infection status antenatally

	All study participants			
	**SARS-CoV-2** **positive**	**SARS-CoV-2** **negative**		
	**n (%)**	**n (%)**	**OR (95% CI)**	aOR* (95% CI)
	**N = 139**	**N = 636**		
Live birth	137 (99)	619 (97)	Reference	Reference
Foetal death	2 (1)	17 (3)	0.53 (0.12-2.33)	0.56 (0.12-2.64)
**Live births overall**				
	**n = 137**	**n = 619**		
Mode of delivery				
Vaginal	59 (43)	277 (45)	Reference	Reference
Caesarean-elective	27 (20)	131 (21)	0.97 (0.59-1.60)	1.11 (0.64-1.93)
Caesarean-emergency	51 (37)	211 (34)	1.13 (0.75-1.72)	0.96 (0.62-1.50)
Apgar score at 5 min				
0-3	1 (1)	2 (0.4)	2.24 (0.20-24.87)	2.28 (0.19-26.92)
4-6	2 (2)	11 (2)	0.81 (0.18-3.72)	0.92 (0.19-4.37)
7-10	126 (98)	564 (98)	Reference	Reference
Respiratory distress				
No	122 (91)	534 (88)	Reference	Reference
Yes	12 (9)	71 (12)	0.74 (0.39-1.41)	0.61 (0.31-1.20)
Gestational age in weeks at delivery				
≥37	99 (73)	489 (79)	Reference	Reference
<37	37 (27)	128 (21)	1.43 (0.93-2.18)	1.28 (0.81-2.02)
Birth weight in grams				
≥2500	94 (70)	466 (78)	Reference	Reference
<2500	40 (30)	133 (22)	1.49 (0.98-2.26)	**1.63 (1.04-2.55)**
<1500	6 (4)	18 (3)	1.65 (0.64-4.28)	1.81 (0.67-4.89)
Preterm/Low-birthweight				
No	85 (62.5)	437 (71)	Reference	Reference
Yes	51 (37.5)	182 (29)	1.44 (0.98-2.12)	1.44 (0.95-2.20)
**Live births, mothers enrolled at <37 weeks gestation**				
	**N = 98**	**N = 450**		
Gestational age in weeks at delivery				
≥37	61 (62)	322 (72)	Reference	Reference
<37	37 (38)	128 (28)	1.53 (0.97-2.41)	1.20 (0.68-2.12)
Birth weight in grams				
≥2500	62 (64)	322 (74)	Reference	Reference
<2500	35 (36)	114 (26)	**1.60 (1.00-2.54)**	1.53 (0.90-2.59)
<1500	6 (6)	18 (4)	1.73 (0.66-4.54)	1.73 (0.61-4.89)
Preterm/Low birthweight				
No	52 (53)	287 (64)	Reference	Reference
Yes	46 (47)	163 (36)	**1.56 (1.00-2.42)**	1.34 (0.79-2.28)
	**Live births mothers enrolled at <34 weeks gestation**			
	**N = 72**	**N = 313**		
Gestational age in weeks at delivery				
≥37	44 (61)	214 (68)	Reference	Reference
<37	28 (39)	99 (32)	1.38 (0.81-2.34)	0.97 (0.45-2.09)
<34	16 (22)	46 (15)	1.69 (0.88-3.26)	0.90 (0.32-2.52)
Birth weight in grams				
≥2500	44 (61)	220 (72)	Reference	Reference
<2500	28 (39)	84 (28)	1.67 (0.97-2.85)	1.61 (0.84-3.08)
<1500	6 (8)	16 (5)	1.88 (0.69-5.06)	1.85 (0.78-5.85)
Preterm/Low birthweight				
No	37 (51)	192 (61)	Reference	Reference
Yes	35 (49)	121 (39)	1.50 (0.90-2.51)	1.26 (0.64-2.51)

OR – odds ratio, aOR – adjusted odds ratio

*Odds ratio adjusted for HIV status (infected, uninfected), comorbid conditions (yes, no), pregnancy related complications (yes, no), previous premature births (yes, no), and time between diagnosis and delivery (categorical variable); for aOR, records numbers are less due to missing data in variables used for adjustment. Odds ratio in bold are statistically significant.

Among women with a negative NAAT result, those presenting with symptoms at enrolment had higher rates of premature birth (aOR = 1.93, 95% CI = 1.13-3.31) compared with asymptomatic women (Table S1 in the **Online Supplementary Document**). No difference in outcomes were detected comparing symptomatic and asymptomatic women at enrolment with a positive NAAT result, although only 19 women had no symptoms (Table S2 in the **Online Supplementary Document**).

Thirty-four of the women enrolled were hospitalised for a respiratory illness at the time of enrolment, of whom 21 (62%) had confirmed COVID-19. Of the women hospitalized with COVID-19, four (19%) were admitted to ICU, two (10%) required ventilation and one demised three days after hospitalization. Post-mortem autopsy confirmed COVID-19 pneumonia as the cause of death in the decedent. Of the 13 women hospitalised with a respiratory illness but tested negative for SARS-CoV-2 infection, none were admitted to ICU or died. One additional death occurred following surgical complications during delivery by Caesarean section in a woman with SARS-CoV-2 which occurred four months prior to delivery.

Placental tissue was available for histological assessment in 35 (31 with COVID-19) and 41 women with and without antenatal SARS-CoV-2 infection, respectively. A positive NAAT result was obtained in 28% (9/32) of the placentas from women with antenatal SARS-CoV-2 infection. The placentas from women with antenatal SARS-CoV-2 infection were more likely to have chorangiosis (OR = 3.40, 95% CI = 1.18-9.84), although not significant in adjusted analysis (OR = 3.01, 95% CI = 0.99-9.17). Maternal vascular malperfusion was more frequently identified in women who tested negative for SARS-CoV-2 antenatally (aOR = 0.28, 95% CI = 0.09-0.89). There were no other histological differences in the placental tissue of women with and without antenatal documented SARS-CoV-2 infection (**Table 4**).

**Table 4:** Placenta histological features by maternal SARS-CoV-2 infection status

	SARS-CoV-2 positive, N = 35, n (%)*	SARS-CoV-2 negative, N = 41, n (%)†	OR (95% CI)	aOR‡ (95% CI)	
**Chorioamnionitis**					
Absent	26 (74)	28 (68)	Reference	Reference	
Present	9 (26)	13 (32)	0.75 (0.27-2.03)	0.64 (0.22-1.85)	
Chronic	2 (6)	1 (2)	2.15 (0.18-25.19)	2.00 (0.16-24.21)	
Acute	7 (20)	12 (29)	0.63 (0.21-1.84)	0.53 (0.17-1.65)	
**Acute chorioamnionitis with maternal and foetal response**					
Absent	4/7 (57)	7/11 (64)	Reference	Reference	
Present	3/7 (43)	4/11 (36)	1.31 (0.19-9.10)	1.17 (0.13-10.52)	
**Funisitis**					
Absent	34 (97)	38 (93)	Reference	Reference	
Present	1 (3)	3 (7)	0.37 (0.04-3.75)	0.27 (0.02-2.98)	
**Chronic villitis**					
Absent	19 (54)	25 (61)	Reference	Reference	
Present	16 (46)	16 (39)	1.32 (0.53-3.28)	1.00 (0.38-2.65)	
High grade	7 (20)	10 (24)	0.92 (0.30-2.87)	0.56 (0.16-1.96)	
Low grade	9 (26)	6 (15)	1.97 (0.60-6.51)	1.85 (0.52-6.60)	
**Histiocytic intervillositis**					
Absent	35 (100)	37 (90)	Reference	Reference	
Present	0 (0)	4 (10)	Na	Na	
**Combination§**					
Absent	35 (100)	40 (98)	Reference	Reference	
Present	0 (0)	1 (2)	Na	Na	
**Meconium laden macrophages**					
Absent	19 (54)	22/40 (55)	Reference	Reference	
Present	16 (46)	18/40 (45)	1.03 (0.41-2.56)	1.10 (0.42-2.89)	
**Maternal vascular malperfusion**				
Absent	25 (71)	21 (51)	Reference	Reference	
Present	10 (29)	20 (49)	0.42 (0.16-1.09)	**0.28 (0.09-0.89)**	
**Foetal vascular malperfusion**					
Absent	20/34 (59)	25/40 (63)	Reference	Reference	
Present	14/34 (41)	15/40 (37)	1.17 (0.46-2.97)	1.16 (0.43-3.10)	
Global	12/34 (35)	14/40 (35)	1.07 (0.41-2.83)	1.22 (0.44-3.34)	
Segmental	2/34 (6)	1/40 (3)	2.50 (0.21-29.60)	0.62 (0.03-12.58)	
**Foetal vascular malperfusion with cord abnormalities**					
Absent	10/14 (71)	10/15 (67)	Reference	Reference	
Present	4/14 (29)	5/15 (33)	0.80 (0.16-3.88)	0.68 (0.06-7.92)	
**Retroplacental hematoma**					
Absent	17 (49)	20 (49)	Reference	Reference	
Present	18 (51)	21 (51)	1.00 (0.41-2.49)	0.88 (0.34-2.32)	
**Foetal compromise**					
**Edematous villi**					
Absent	22/34 (65)	29 (71)	Reference	Reference	
Present	12/34 (35)	12 (29)	1.32 (0.50-3.49)	1.67 (0.59-4.71)	
**Increased foetal nucleated red blood cells**					
Absent	32/34 (94)	40 (98)	Reference	Reference	
Present	2/34 (6)	1 (2)	2.50 (0.22-28.83)	1.10 (0.06-19.35)	
**Chorangiosis**					
Absent	20/34 (59)	34 (83)	Reference	Reference	
Present	14/34 (41)	7 (17)	**3.40 (1.18-9.84)**	3.01 (0.99-9.17)	

OR – odds ratio, aOR – adjusted odds ratio, Na – not applicable

*Includes 4 asymptomatic women at the time of enrolment and 31 presenting with symptoms. SARS-CoV-2 nucleic acid amplification test was performed in 32 placentas and was positive in nine.

†Includes 30 asymptomatic women at the time of enrolment and 11 presenting with symptoms.

‡Odds ratio adjusted for HIV status (infected, uninfected), comorbid conditions (yes, no), pregnancy related complications (yes, no).

§Combination of high grade lymphocytic and histiocytic villitis and histiocytic intervillositis and diffuse perivillous fibrin. Odds ratio in bold are statistically significant.

## DISCUSSION

In this prospective study, we report that antenatal SARS-CoV-2 infection mostly detected in the third trimester of pregnancy, was associated with 1.6-higher odds of having a low-birthweight newborn. A similar observation was evident when restricting the analysis to symptomatic infections, ie, COVID-19 cases. Although most of the participants in our study had mild disease, 21 of the SARS-CoV-2 infected women were hospitalized – among whom one died due to COVID-19 pneumonia.

A meta-analysis of studies up to June 30, 2021 reported that pregnant women with COVID-19 compared to those without had 1.69-higher odds of having a low-birthweight newborn (based on 3 studies with 807 and 1743 women, respectively), 1.86-higher odds of preterm birth (18 studies including 10 555 and 498 064 women, respectively), 1.46-higher odds of stillbirth (based on 6 studies with 8392 and 487 395 women, respectively) and 1.44-higher odds of 5th minute Apgar score of less than 7 (based on 4 studies with 2777 and 88 909 women, respectively); although symptoms at the time of testing and timing of SARS-CoV-2 infection were not described for these outcomes [15].

As previously reported, we confirmed that SARS-CoV-2 could be associated with asymptomatic infections [21,26]. When looking at symptoms presentation the INTERCOVID Multinational Cohort Study, conducted in 18 countries among 706 pregnant women with and 1424 without SARS-CoV-2 infection at any stage of pregnancy or delivery, reported a trend for earlier gestational age at delivery in women with symptomatic compared with asymptomatic SARS-CoV-2 infection [8]. Also, in a Spanish population-based study even though no differences were found in the overall rate of adverse outcomes among SARS-CoV-2 infected and uninfected pregnant women; those with symptomatic COVID-19 had increased rates of preterm delivery (7.2% vs 16.9%) and intrapartum foetal distress (9.1% vs 19.2%) compared with SARS-CoV-2 uninfected women. Women with asymptomatic infection, however, had similar rates of preterm delivery compared with women who had not been infected by SARS-CoV-2 [27]. In contrast, the frequency of preterm birth (13%) among pregnant women in the USA with SARS-CoV-2 infection was similar in those presenting with symptoms (2315 women) compared with asymptomatic women (376 women) in unadjusted analyses [28]. Our study results underscore the importance to stratify SARS-CoV-2 infections into symptomatic (COVID-19) and asymptomatic illness and the contribution of symptoms due to other causes. Notably, we also observed a higher frequency of preterm deliveries among women with negative SARS-CoV-2 NAAT results in those with compared to those without respiratory symptoms, which could have been due to other respiratory pathogens infections. Notably, however, seasonal influenza virus was not circulating during the time period of our study [29].

There have been few reports on placental pathology in mothers who acquired SARS-CoV-2 during pregnancy describing a spectrum of features from normal tissue to diffuse inflammatory patterns in the intervillous space [30-32]. The lack of conformity in the period between acquiring the virus to delivery, maternal comorbidities and variability in the laboratory methods used to confirm infection, however, limit interpretation of the current data. In our study, we identified higher prevalence of chorangiosis in the placentas of women with documented SARS-CoV-2 infection. Chorangiosis in a nonspecific adaptive placental response to decreased maternal oxygen tension, usually chronic or prolonged and has been previously described in the placentas from mothers with anaemia, diabetes, umbilical cord obstruction, or who smoke, or who live at very high altitudes [33,34]. It has also been reported among COVID-19 cases [35]. Maternal vascular malperfusion, although detected on both positive and negative NAAT groups, was more frequently identified in those women without history of SARS-CoV-2 infection. The women included in our study, done at two secondary and tertiary hospitals, had many other comorbidities including HIV infection. We found that >40% of the women had pregnancy-related complications, which can independently result in placental changes. The inclusion of a SARS-CoV-2 uninfected group is crucial to describe the specific effect that SARS-CoV-2 infection has on the placenta.

A limitation of our study was that antenatal testing was performed only once during pregnancy and we cannot exclude the possibility that women who had a negative NAAT result at time of enrolment may not have acquired an infection at a later stage. Also, since gestational sonar was not performed as part of the study and not done routinely, we relied on the date of the last menstrual period assessment documented in antenatal and medical notes to assess gestational age, which may lead to inaccurate gestational age staging [36]. Therefore, we used low and very low birthweight as a proxy for preterm birth. This was an observational study and therefore subject to potential biases and confounding effects, although we adjusted the outcome analyses for HIV infection status, other comorbid conditions and pregnancy-related complications, we did not evaluate the full impact of these variables.

Our study demonstrates that in a setting with high HIV infection prevalence and other comorbidities antenatal SARS-CoV-2 infection was associated with preterm delivery. Another study in South Africa, also during the first pandemic wave (May to July 2020), among women with high-risk pregnancies with COVID-19, found high rates (40%) of severe COVID-19 but no differences in disease severity or pregnancy outcomes in women living with HIV compared with pregnant women with other conditions [37]. A retrospective analysis of pregnant women from six African countries found that the frequencies of preterm births and low-birthweight infants were similar between 213 women with and 302 without COVID-19, however this cohort only included hospitalized women and so the control group was heterogeneous with a range of diseases that could have be associated with adverse pregnancy outcomes [11].

## CONCLUSIONS

More studies are needed from low- and middle-income countries to understand the possible impact of other comorbidities in pregnancy outcomes, since it has been suggested that pregnant women and their neonates from low- and middle-income countries are more vulnerable to adverse outcomes due to COVID-19 than women from high income countries [38]. Vaccination of pregnant women with COVID-19 vaccines has been shown to induce a good immune response, with efficient transplacental transfer of binding antibody to the foetus [39]. Also, COVID-19 vaccination of pregnant women reduces the risk of COVID-19 in the women and their young infants; and has also been reported to lower the risk of stillbirths [40]. Therefore, COVID-19 vaccination should be encouraged in pregnant women.

## Additional material:


Online Supplementary Document
