# Supplementary material for: Antepartum SARS-CoV-2 infection and adverse birth outcomes in South African women
Source: J Glob Health. 2022 Dec 3;12:05050. doi: 10.7189/jogh.12.05050 (PMC9718447; doi:10.7189/jogh.12.05050)
Supplement: Online Supplementary Document [file jogh-12-05050-s001.pdf]

ONLINE SUPPLEMENTARY DOCUMENT

Antepartum SARS-CoV-2 infection and adverse birth outcomes in South African women

Marta C Nunes, Stephanie Jones, Renate Strehlau, Vuyelwa Baba, Zanele Ditse, Kelly da Silva, Lané Bothma, Natali Serafin, Vicky L Baillie, Gaurav Kwatra, Megan Burke, Amy Wise, Mary Adam, Philiswa Mlandu, Mpolokeng Melamu, Juliette Phelp, Wendy Fraser, Colleen Wright, Elizabeth Zell8, Yasmin Adam, Shabir A Madhi

Placenta Pathology Assessment

Diagnosis of maternal vascular malperfusion (MVM) was based on a constellation of macroscopic and microscopic findings, approximately 9-12 in total, not all of which were present in an individual case. *Macroscopic findings* included low placental weight (for gestational age), infarction and retroplacental haemorrhage. The placenta was considered small (placental hypoplasia) if the weight was <10th centile for the gestational age. Macroscopic infarction was defined as firm solid areas extending from the basal plate with the apex towards the foetal surface. Any infarction in a preterm placenta and >5% infarction at term was determined pathological. *Microscopic features* of MVM included microscopic infarction, distal villous hypoplasia, accelerated villous maturation, decidual arteriopathy and increased syncytial knots. Increased perivillous fibrin, previously a feature of MVM, is now no longer part of the Amsterdam consensus criteria for MVM (1) but was still recorded in this study as diffuse perivillous fibrin is in its own right a pathological entity of clinical significance (2). Microscopic infarction was diagnosed as villi crowded together (in contrast to perivillous fibrin where the fibrin pushes the villi apart). In recent infarcts the villi may be congested while in old infarction they are avascular with infiltration of neutrophils and fibrin. Distal villous hypoplasia was diagnosed when villi were small and stringy, elongated or appeared as tiny, barely the size of a syncytial knot. It may be focal or diffuse and is usually only seen in placentas earlier than 32 weeks, indicating early onset MVM. Accelerated villous maturation indicates villi hyper mature for a known gestational age and is usually associated with an increase in syncytial knots. Decidual arteriopathy indicates an absence of spiral arterial remodelling with mural hypertrophy only, or with fibrinoid necrosis with foam cells. Increased syncytial knots were reported if they were present in >33% of villi at term.

Diagnosis of Foetal Vascular Malperfusion (FVM) was based on avascular villi, thrombosis in chorionic plate and/or stem villous vessels, intramural fibrin deposition and villous-stromal karyorrhexis. Villous stromal karyorrhexis, previously called haemorrhagic endovasculitis, was defined as regional or diffuse,

and demonstrates nuclear dust with extravasation of red cells into the villous stroma with preservation of the surrounding trophoblast. FVM may be global (partial umbilical cord obstruction) or segmental (complete) and either may be high grade or low grade.

Abruption is a clinical diagnosis, but the corresponding pathological term is retroplacental haemorrhage. Macroscopic retroplacental haemorrhage was defined as an indentation (with or without adherent clot) of >15% of the maternal surface of the placenta, possibly with bleeding seen tracking into the placental parenchyma after the placenta is sliced and the slices are laid out flat on the surface of the cutting table. The microscopic criteria for retroplacental haemorrhage were the presence of blood beneath the decidua, dissecting into the decidua and placental parenchyma, with congestion, and/or intravillous haemorrhage (haemorrhage into villous stroma). There may be additional coagulation necrosis of the syncytiotrophoblast nuclei with overlying infarction.

Features of infection included chorioamnionitis (ascending infection), and villitis which was divided into infectious and non-infectious chronic villitis or villitis of unknown aetiology.

Ascending infection or chorioamnionitis was defined as neutrophils in the placenta - maternal response- with or without the involvement of the umbilical cord vessels (vein and arteries or funisitis). The location of the inflammation within the membranes of the chorionic plate, and additional features such as subchorionic micro-abscesses were recorded.

Villitis was recorded as acute (extremely uncommon) or chronic.If features such as plasma cells, granulomas, multinucleated giant cells, normoblasts in foetal vessels and hemosiderin within sclerotic villi or an accompanying necrotising funisitis were present, further investigations were undertaken to exclude a specific infective aetiology such as cytomegalovirus, Treponema pallidum or toxoplasmosis (3). The remainder were designated as chronic non-specific villitis or villitis of unknown aetiology, a maternal T-lymphohistiocytic response to foetal antigens. These were graded as high grade or low grade and the presence of obliterative foetal vasculopathy noted, as it has prognostic significance (4). The cell type was noted as lymphocytic, histiocytic or a combination of both.

The association of chronic lymphohistiocytic villitis with chronic histiocytic villitis and perivillous fibrin was specifically noted in view of the current literature described in patients with COVID-19.

Chronic Histiocytic Intervillositis is an infiltrate of the intervillous space or maternal blood by CD68+ histiocytes, with or without accompanying fibrin, and rarely with a minor component of chronic villitis.

Amnion nodosum is deposition of foetal squames and vernix on the membranes, usually associated with severe oligohydramnios. It may be a marker for pulmonary hypoplasia and renal abnormalities in the foetus.

Further pathological lesions indicative of non-specific placental injury/foetal compromise recorded were villous edema (focal or diffuse), increased foetal nucleated red blood cells, and chorangiosis (focal and diffuse).

Table S1: Pregnancy outcomes among women enrolled antenatally without a SARS-CoV-2 positive test, by symptoms presentation

	**All antenatal SARS-CoV-2 negative test study participants**					
	**Symptomatic**		**Asymptomatic**			
	**N=151**		**N=484**			
	**n**	**(%)**	**n**	**(%)**	**OR (95% CI)**	**aOR[1] (95% CI)**
Live birth	145	(96)	473	(98)	Ref	Ref
Foetal death	6	(4)	11	(2)	1.78 (0.65, 4.89)	2.33 (0.72, 7.51)
	**Live births overall**					
	**N=145**		**N=473**			
	**n**	**(%)**	**n**	**(%)**	**OR (95% CI)**	**aOR[1] (95% CI)**
Gestational age in weeks at delivery						
≥37	99	(69)	388	(82)	Ref	Ref
<37	44	(31)	85	(18)	**2.03 (1.33, 3.11)**	**1.93 (1.20, 3.11)**
Birthweight in grams						
≥2500	101	(73)	364	(79)	Ref	Ref
<2500	38	(27)	95	(21)	1.44 (0.93, 2.23)	1.21 (0.74, 2.00)
<1500	9	(6)	9	(2)	**3.60 (1.39, 9.32)**	2.68 (0.93, 7.70)
Preterm/Low-birthweight						
No	86	(59)	347	(73)	Ref	Ref
Yes	59	(41)	126	(27)	**1.89 (1.28, 2.79)**	**2.00 (1.28, 3.11)**
	**Live births, mothers enrolled at <37 weeks gestation**					
	**N=115**		**N=335**			
	**n**	**(%)**	**n**	**(%)**	**OR (95% CI)**	**aOR[1] (95% CI)**
Gestational age in weeks at delivery						
≥37	71	(62)	250	(75)	Ref	Ref
<37	44	(38)	85	(25)	**1.82 (1.16, 2.86)**	**1.93 (1.13, 3.31)**
Birthweight in grams						
≥2500	78	(70)	244	(75)	Ref	Ref
<2500	33	(30)	84	(25)	1.28 (0.79, 2.06)	0.98 (0.56, 1.70)
<1500	9	(8)	9	(3)	**3.13 (1.20, 8.16)**	2.28 (0.78, 6.66)
Preterm/Low-birthweight						
No	61	(53)	224	(67)	Ref	Ref

Yes	54	(47)	111	(33)	**1.79 (1.16, 2.75)**	**1.93 (1.15, 3.22)**
	**Live births, mothers enrolled at <34 weeks gestation**					
	**N=84**		**N=229**			
	**n**	**(%)**	**n**	**(%)**	**OR (95% CI)**	**aOR[1] (95% CI)**
Gestational age in weeks at delivery						
≥37	52	(62)	161	(70)	Ref	Ref
<37	32	(38)	68	(30)	1.46 (0.86, 2.46)	1.65 (0.85, 3.21)
<34	14	(17)	32	(14)	1.35 (0.81, 2.95)	1.82 (0.45, 3.90)
Birthweight in grams						
≥2500	58	(71)	162	(73)	Ref	Ref
<2500	24	(29)	60	(27)	1.12 (0.64, 1.96)	0.81 (0.41, 1.59)
<1500	7	(9)	9	(4)	2.17 (0.77, 6.10)	1.62 (0.46, 5.68)
Preterm/Low-birthweight						
No	46	(55)	144	(63)	Ref	Ref
Yes	38	(45)	85	(37)	1.40 (0.84, 2.32)	1.50 (0.80, 2.83)

[1]Odds Ratio (aOR) adjusted for HIV status (infected, uninfected), comorbid conditions (yes, no), pregnancy related complications (yes, no), previous premature births (yes, no), and time between diagnosis and delivery (categorical variable); for aOR, records numbers are less due to missing data in variables used for adjustment.

Odds Ratio in bold are statistically significant.

Supplementary Table 2: Pregnancy outcomes among women enrolled antenatally with a SARS-CoV-2 positive test, by symptoms presentation

	All NAAT-positive study participants					
	**Symptomatic**		**Asymptomatic**			
	**N=119**		**N=19**			
	**n**	**(%)**	**n**	**(%)**	**OR (95% CI)**	**aOR[1] (95% CI)**
Live birth	117	(98)	19	(100)		
Foetal death	2	(2)	0	(0)	P=0.99	
	**Live births overall**					
	**N=117**		**N=19**			
	**n**	**(%)**	**n**	**(%)**	**OR (95% CI)**	**aOR[1] (95% CI)**
Gestational age in weeks at delivery						
≥37	85	(74)	12	(63)	Ref	Ref
<37	30	(26)	7	(37)	0.61 (0.22, 1.68)	0.76 (0.24, 2.41)
Birthweight in grams						
≥2500	80	(70)	13	(68)	Ref	Ref
<2500	34	(30)	6	(32)	0.92 (0.32, 2.62)	0.95 (0.29. 3.05)
<1500	6	(5)	0	(0)	P=0.99	-
Preterm/Low-birthweight						
No	72	(63)	11	(56)	Ref	Ref
Yes	43	(37)	8	(42)	0.82 (0.31, 2.20)	0.97 (0.33, 2.91)
	**Live births, mothers enrolled at <37 weeks gestation**					
	**N=85**		**N=13**			
	**n**	**(%)**	**n**	**(%)**	**OR (95% CI)**	**aOR[1] (95% CI)**
Gestational age in weeks at delivery						
≥37	53	(64)	6	(46)	Ref	Ref
<37	30	(36)	7	(54)	0.49 (0.15, 1.58)	0.48 (0.12, 2.01)
Birthweight in grams						
≥2500	53	(64)	8	(62)	Ref	Ref
<2500	30	(36)	5	(38)	0.91 (0.27, 3.02)	1.01 (0.23, 4.40)
<1500	6	(7)	0	(0)	P=0.70	-
Preterm/Low-birthweight						
No	44	(53)	6	(46)	Ref	Ref

	**All NAAT-positive study participants**					
Yes	39	(47)	7	(54)	0.76 (0.24, 2.45)	0.94 (0.23, 3.81)
	**Live births, mothers enrolled at <34 weeks gestation**					
	**N=63**		**N=9**			
	**n**	**(%)**	**n**	**(%)**	**OR (95% CI)**	**aOR[1] (95% CI)**
Gestational age in weeks at delivery						
≥37	38	(61)	5	(56)	Ref	Ref
<37	24	(39)	4	(44)	0.79 (0.19, 3.24)	0.66 (0.12, 3.56)
<34	13	(21)	3	(33)	0.57 (0.12, 2.72)	0.50 (0.07, 3.41)
Birthweight in grams						
≥2500	37	(60)	6	(67)	Ref	Ref
<2500	25	(40)	3	(33)	1.35 (0.31, 5.91)	2.34 (0.36, 15.36)
<1500	6	(10)	0	(0)	P=0.99	-
Preterm/Low-birthweight						
No	31	(50)	5	(56)	Ref	Ref
Yes	31	(50)	4	(44)	1.25 (0.31, 5.10)	1.59 (0.29, 8.78)

[1]Odds Ratio (aOR) adjusted for HIV status (negative, positive), comorbid conditions (yes, no) and pregnancy related complications (yes, no); for aOR, records numbers are less due to missing data in variables used for adjustment.
Odds Ratio in bold are statistically significant.

References:
1. Khong TY, Mooney EE, Ariel I, Balmus NCM, Boyd TK, Brundler M-A, et al. Sampling and Definitions of Placental Lesions. Archives of Pathology & Laboratory Medicine. 2016;140(7):698-713.
2. Chen A, Roberts DJ. Placental pathologic lesions with a significant recurrence risk – what not to miss! APMIS. 2018;126(7):589-601.
3. Al-Adnani M, Sebire NJ. The role of perinatal pathological examination in subclinical infection in obstetrics. Best Practice & Research Clinical Obstetrics & Gynaecology. 2007;21(3):505-21.
4. Katzman P. Chronic inflammatory lesions of the placenta. Seminars in Perinatology. 2015;39:20-6.
